# Supplementary figures and images for: Seasonal variation and group size affect movement patterns of two pelagic dolphin species (Lagenorhynchus obscurus and Delphinus delphis)
Source: PLoS One. 2022 Nov 9;17(11):e0276623. doi: 10.1371/journal.pone.0276623 (PMC9645598; doi:10.1371/journal.pone.0276623)

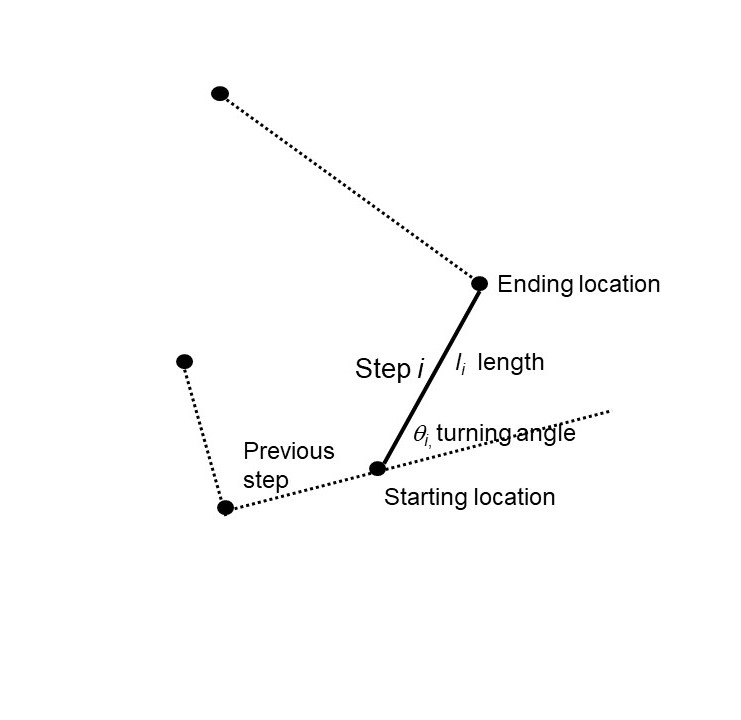

Supplement: S1 Fig — (JPG) [file pone.0276623.s001.jpg]

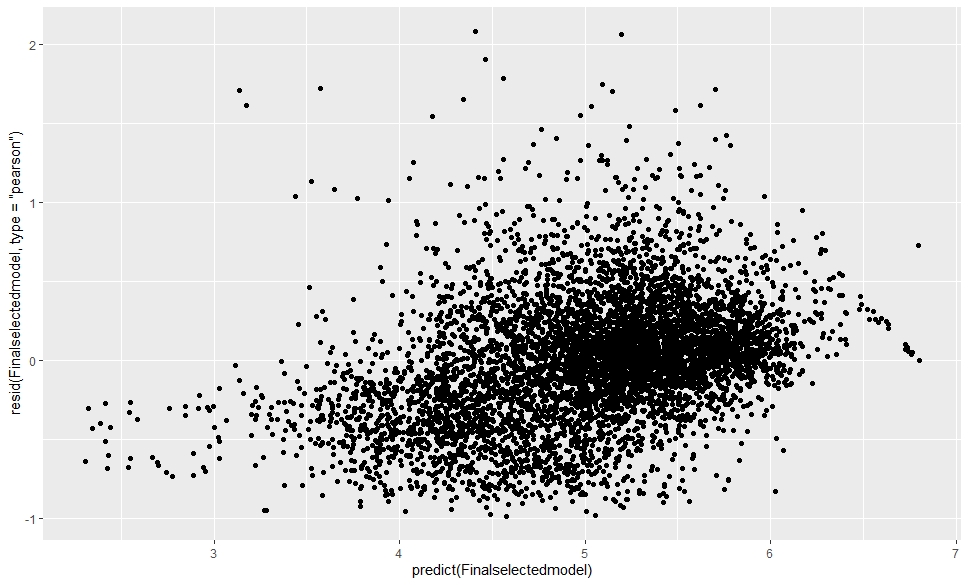

Supplement: S2 Fig — (TIF) [file pone.0276623.s002.tif]
