# Supplementary material for: Seasonal variation and group size affect movement patterns of two pelagic dolphin species (Lagenorhynchus obscurus and Delphinus delphis)
Source: PLoS One. 2022 Nov 9;17(11):e0276623. doi: 10.1371/journal.pone.0276623 (PMC9645598; doi:10.1371/journal.pone.0276623)
Supplement: S1 File — (DOCX) [file pone.0276623.s003.docx]

**Supplementary Information**

**S1 Table. Evaluation of the random term in the model structure. Models were estimated with REML.**

| **Model structure** | **ΔAIC** | **df** |
| --- | --- | --- |
| Gamma with random effect term | 0.0 | 49 |
| Gamma without random effect term | 22.1 | 48 |
| Gaussian with random effect term | 1023.2 | 49 |
| Gaussian without random effect term | 1150.7 | 48 |

**S2 Table. Regression coefficients for all the terms in the final selected model. The estimated value, 2.5% bound, 97.5% bound, z-value, and p-value are shown. The bounds were computed based on likelihood profiles.**

|  | **Estimate** | **2.5% bound** | **97.5% bound** | **z value** | **Pr(>\|z\|)** |
| --- | --- | --- | --- | --- | --- |
| (Intercept) | 4.77 | 4.51 | 5.03 | 35.84 | < 2e 16 *** |
| BehaviorOther | 0.02 | -0.20 | 0.24 | 0.19 | 0.8467 |
| BehaviorTraveling | 0.05 | -0.19 | 0.28 | 0.39 | 0.6934 |
| Species | 0.17 | -0.13 | 0.44 | 1.09 | 0.2739 |
| TurningAngle | 0.20 | 0.16 | 0.24 | 9.66 | < 2e 16 *** |
| Season | -0.33 | -0.45 | -0.20 | 5.19 | 2.07e 07 *** |
| Group size>100 | 0.69 | 0.16 | 1.22 | 2.55 | 0.0108 * |
| Group size 10 20 | -0.08 | -0.43 | 0.28 | 0.43 | 0.6703 |
| Group size20 50 | 0.28 | -0.12 | 0.67 | 1.36 | 0.1730 |
| Group size 50 70 | 0.56 | -0.07 | 1.20 | 1.74 | 0.0817 . |
| Group size 70 100 | 0.48 | -0.14 | 1.11 | 1.52 | 0.1283 |
| BehaviorOther;Species | -0.03 | -0.26 | 0.20 | 0.26 | 0.7946 |
| BehaviorTraveling:Species | 0.20 | -0.03 | 0.44 | 1.71 | 0.0880 . |
| TurningAngle:Season | -0.13 | -0.20 | -0.06 | 3.55 | 0.0004 *** |
| BehaviorOther:GroupSize>100 | 0.21 | -0.04 | 0.46 | 1.67 | 0.0958 . |
| BehaviorTraveling:GroupSize>100 | 0.31 | 0.07 | 0.56 | 2.48 | 0.0132 * |
| BehaviorOther:GroupSize10 20 | 0.21 | -0.02 | 0.43 | 1.76 | 0.0789 . |
| BehaviorTraveling:GroupSize10 20 | 0.26 | 0.03 | 0.50 | 2.23 | 0.0257 * |
| BehaviorOther:GroupSize20 50 | -0.07 | -0.30 | 0.16 | 0.60 | 0.5508 |
| BehaviorTraveling:GroupSize20 50 | -0.01 | -0.24 | 0.22 | 0.08 | 0.9356 |
| BehaviorOther: GroupSize50 70 | 0.23 | -0.07 | 0.53 | 1.51 | 0.1307 |
| BehaviorTraveling: GroupSize50 70 | 0.08 | -0.19 | 0.35 | 0.59 | 0.5566 |
| BehaviorOther: GroupSize70 100 | 0.07 | -0.30 | 0.44 | 0.37 | 0.7109 |
| BehaviorTraveling: GroupSize70 100 | 0.34 | 0.03 | 0.64 | 2.13 | 0.0329 * |
| Species: GroupSize >100 | -0.87 | -1.40 | -0.35 | 3.26 | 0.0011 ** |
| Species: GroupSize10 20 | -0.07 | -0.41 | 0.27 | 0.40 | 0.6887 |
| Species: GroupSize20 50 | -0.20 | -0.59 | 0.19 | 1.01 | 0.3135 |
| Species: GroupSize50 70 | -0.53 | -1.15 | 0.09 | 1.69 | 0.0915 . |
| Species: GroupSize70 100 | -0.74 | -1.38 | -0.10 | 2.28 | 0.0227 * |
